# Incidence and risk factors of medical complications and direct medical costs after osteoporotic fracture among patients in China

**DOI:** 10.1007/s11657-018-0429-5

**Published:** 2018-02-27

**Authors:** Ruiqi Liu, Aijun Chao, Ke Wang, Jing Wu

**Affiliations:** 1 School of Pharmaceutical Science and Technology, Tianjin University, No. 92 Weijin Rd., Nankai District, Tianjin, 300072 China; 2 Tianjin Hospital, Tianjin, 300072 China; 3 Lilly Suzhou Pharmaceutical Co. Ltd., Shanghai, 200021 China

**Keywords:** Osteoporotic fracture, Hip fracture, Vertebral fracture, Complications, Risk factors, Economic burden

## Abstract

**Summary:**

We analyzed the incidence of medical complications after osteoporotic fractures and estimated its risk factors and cost impacts. Osteoporotic fractures can result in lots of serious medical complications, which is associated with patients’ baseline characteristics such as patients’ disease history and significantly increased patients’ direct medical costs.

**Purpose:**

The purpose of the study is to investigate the incidence and identify the risk factors of medical complications after osteoporotic fracture, and quantify patients’ economic burden.

**Methods:**

Data were obtained from the Tianjin Urban Employee Basic Medical Insurance database (2009–2014). Patients aged ≥ 50 years, had ≥ 1 diagnoses of hip or vertebral fracture between 2010 and 2012, and continuously enrolled from 12 months before to 24 months after the first identified fracture were included. The incidence of medical complications was estimated within 12 months before and after fracture. Direct medical costs were measured and compared between patients with at least one medical complication and without any medical complications. Logistic regression was applied to identify risk factors for any medical complications.

**Results:**

Three thousand seven hundred nineteen patients were identified; 45.0% had hip fracture, and 56.2% had vertebral fracture. After osteoporotic fracture, the accumulative incidence of the most common medical complications including constipation (25.6%, RR 1.38 [1.28, 1.48]), stroke (25.2%, 1.16 [1.09, 1.24]), pneumonia (17.0%, 1.55 [1.40, 1.73]), urinary tract infection (16.3%, 1.23 [1.12, 1.36]), and arrhythmia (11.8%, 1.39 [1.23, 1.56]) was significantly higher than that before fracture. Advanced age; male sex; retirement status; diagnosis of hypertension, chronic heart disease, cerebrovascular disease, hemiplegia, or Parkinson’s disease; and higher direct medical costs at baseline were significant predictors of complications. The all-cause direct medical cost during 24-month follow-up was $5665. Medical complications significantly increased patients’ direct medical costs.

**Conclusions:**

Osteoporotic fractures led to amount of medical complications, which significantly increased patients’ economic burden. Complications correlate to various factors such as patients’ disease history.

**Electronic supplementary material:**

The online version of this article (10.1007/s11657-018-0429-5) contains supplementary material, which is available to authorized users.

## Introduction

Osteoporosis is the most common systemic and metabolic skeletal disease, characterized by low bone mass, deterioration of bone tissue, disruption of bone architecture, compromised bone strength, and increased risk of fracture [1]. Osteoporotic fragility fractures, predominantly vertebral, hip, proximal humeral, and distal forearm fractures, are clinical consequences of osteoporosis [1]. Approximately 40–50% of females and 13–22% of males sustained at least one osteoporotic fracture over the course of their entire life [2]. The number of patients with any osteoporotic fracture in China in 2010 was 2.33 million, a number that is projected to increase to 5.99 million by 2050 [3].

Previous studies have indicated that osteoporotic fractures are significantly associated with a higher risk of subsequent fracture and excess mortality [4, 5]. The accumulative incidence of subsequent fracture within 5 years after an initial osteoporotic fracture was 24% in females and 20% in males, whereas the 5-year accumulative all-cause mortality rate was 24% in females and 27% in males, respectively [4]. Furthermore, fragility fractures could also significantly lower patients’ health-related quality of life, and this loss was sustained for at least 18 months for hip and vertebral fractures [5].

In addition, the economic strain on patients and the healthcare system resulting from osteoporotic fractures are heavy. The relevant annual treatment costs of osteoporotic fracture were calculated at $9.45 billion, and it will increase to roughly $25.43 billion by 2050 in China [3]. One study conducted in western China indicated that the average direct medical cost for osteoporotic fracture patients was approximately RMB 17007 ($2699) per year per patient; the economic burden attributed to hip fracture was highest, followed by vertebral fracture [6].

Previous studies showed that osteoporotic fractures are consistently followed by a large number of medical complications, such as cardiac diseases, venous thromboembolism, pneumonia, urinary tract complications, gastrointestinal tract bleeding, and fluid/electrolyte abnormalities [7–10]. Published studies have shown that general complications following any osteoporotic fracture were markedly associated with a loss of function and can notably affect functional outcomes, resulting in poor prognosis and treatment satisfaction [11, 12]. Fracture patients with complications after a primary fracture always had higher mortality than those without complications. For instance, Roche et al. [13] demonstrated that the mortality at 30 days among hip fracture patients with postoperative heart failure or chest infection was 16.1-fold and 8.5-fold, respectively, compared with those without postoperative heart failure or chest infection. Panagiota et al. [14] pointed out that complications can dramatically extend patients’ length of stay and increase the economic burden related to fracture accordingly.

There has been research assessing the incidence of complications after an osteoporotic fracture [8, 10, 15–17]. However, these studies, especially those in Chinese patients, either considered only complications resulting from fracture surgeries and not those related to conventional treatments or were based on small sample sizes that impacted the generalizability of their results [15–17]. Moreover, no studies thus far have quantified the influence of medical complications on their economic burden in mainland China. Hence, this retrospective database cohort study focused on a broader set of complications and evaluated the associated economic burden in China. The osteoporotic fractures in this study included hip and vertebral fractures because they are the two most serious and commonly occurring osteoporotic fractures [5, 6].

This study aimed to (1) investigate the incidence of medical complications following an osteoporotic fracture and identify its risk factors and (2) estimate and quantify the related direct medical costs of osteoporotic fracture patients.

## Methods

### Data sources

This retrospective cohort study was based on data extracted from the Tianjin Urban Employee Basic Medical Insurance (UEBMI) (2009–2014), one of the three basic medical insurance systems in China covering all employees and retirees in all public and private sectors. By 2014, there were 5.10 million enrollees in the Tianjin UEBMI database, accounting for 33.6% of resident population of Tianjin in 2014 [18]. This study was based on a 30% random sample from the Tianjin UEBMI. The UEBMI database includes all the claims of enrollees in all inpatient, outpatient, and pharmacy services. The detailed claim information included in this database contains desensitized registration files of enrollees’ eligibility, medical service utilization, and fee-for-service claims, including patient unique identification number, demographic information (age, sex, working status), disease diagnoses, healthcare resource utilization (outpatient, inpatient, and pharmacy), and direct medical costs. The disease diagnoses in this database are prescribed using International Statistical Classification of Diseases and Related Health Problems, 10th revision (ICD-10) codes and Chinese description words of osteoporotic fracture. Direct medical costs of enrollees include all the expenditures related to drugs, medical consumables, physical and biochemical tests, non-medication treatments, surgeries, bed fees, and other services (e.g., blood transfusion).

### Sample selection

Considering that osteoporotic fracture mostly affects postmenopausal women and elderly men, the study population was defined as females and males aged ≥ 50 years in Tianjin, China. Patients aged ≥ 50 years, diagnosed with at least one hip or vertebral fracture from January 1, 2010 to December 31, 2012, and continuously enrolled for 12 months before and 24 months after the diagnosis date of the first identified osteoporotic fracture were included in this study. Hip and vertebral fractures were identified using ICD-10 codes (S72x for hip fracture and S12.0–S12.6, S22.0, S22.2, S22.9, and S32.0–S32.5 for vertebral fracture) combined with Chinese description words. To minimize the probability that a normal follow-up medical service visit for a previous osteoporotic fracture would be selected as a new fracture, patients with any fracture history before the index osteoporotic fracture were excluded from this study. Patients diagnosed with cancer, multiple myeloma, or Paget’s disease of bone, which can seriously affect patients’ physical condition, during the entire study time horizon were also excluded. The diagnosis date of the first identified osteoporotic fracture was defined as the index date; the 12 months before the index date was defined as the baseline period, and the 24 months after the index date was defined as the follow-up period.

### Main outcome measures

Consistent with the purposes described above, this study has three primary outcomes: (1) the accumulative incidence of medical complications within 12 months before and after the index date, (2) the risk factors of medical complications, and (3) the direct medical costs incurred within the 12- and 24-month follow-up periods.

**Fig. 1:**
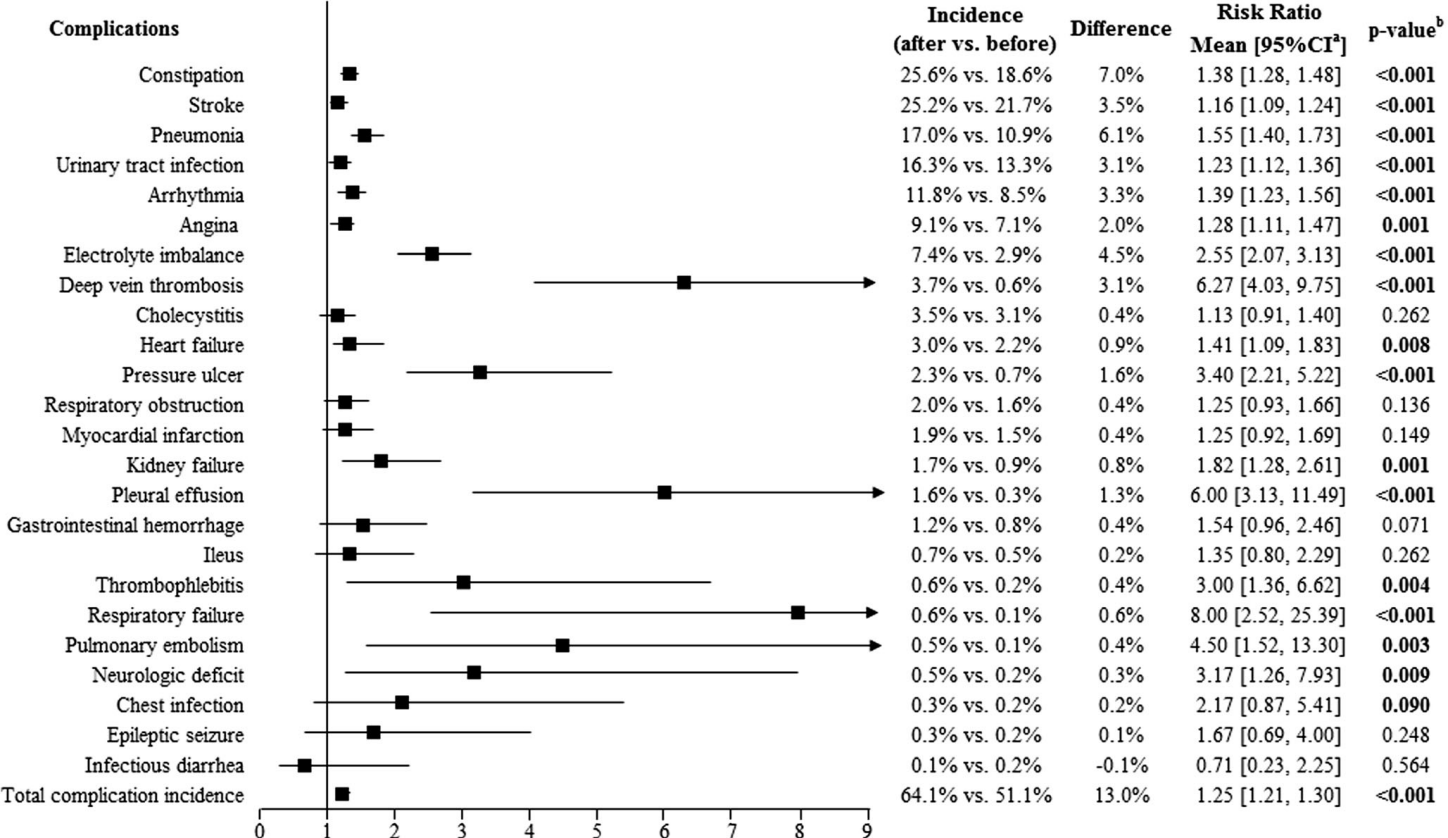
Accumulative incidence of medical complications within 12 months before and after fracture among total osteoporotic fracture patients (*N* = 3719). The accumulative incidence of temporary respiratory insufficiency, diverticulitis, pulmonary edema, and infection of the central venous catheter was equal to zero, both in the 12 months before and after the index date. Difference = the incidence of complications within 12 months after fracture − the incidence of complications with 12 months before fracture. ^a^95% confidence interval (CI) of the risk ratio. ^b^Calculated using McNemar’s chi-squared test

**Fig. 2:**
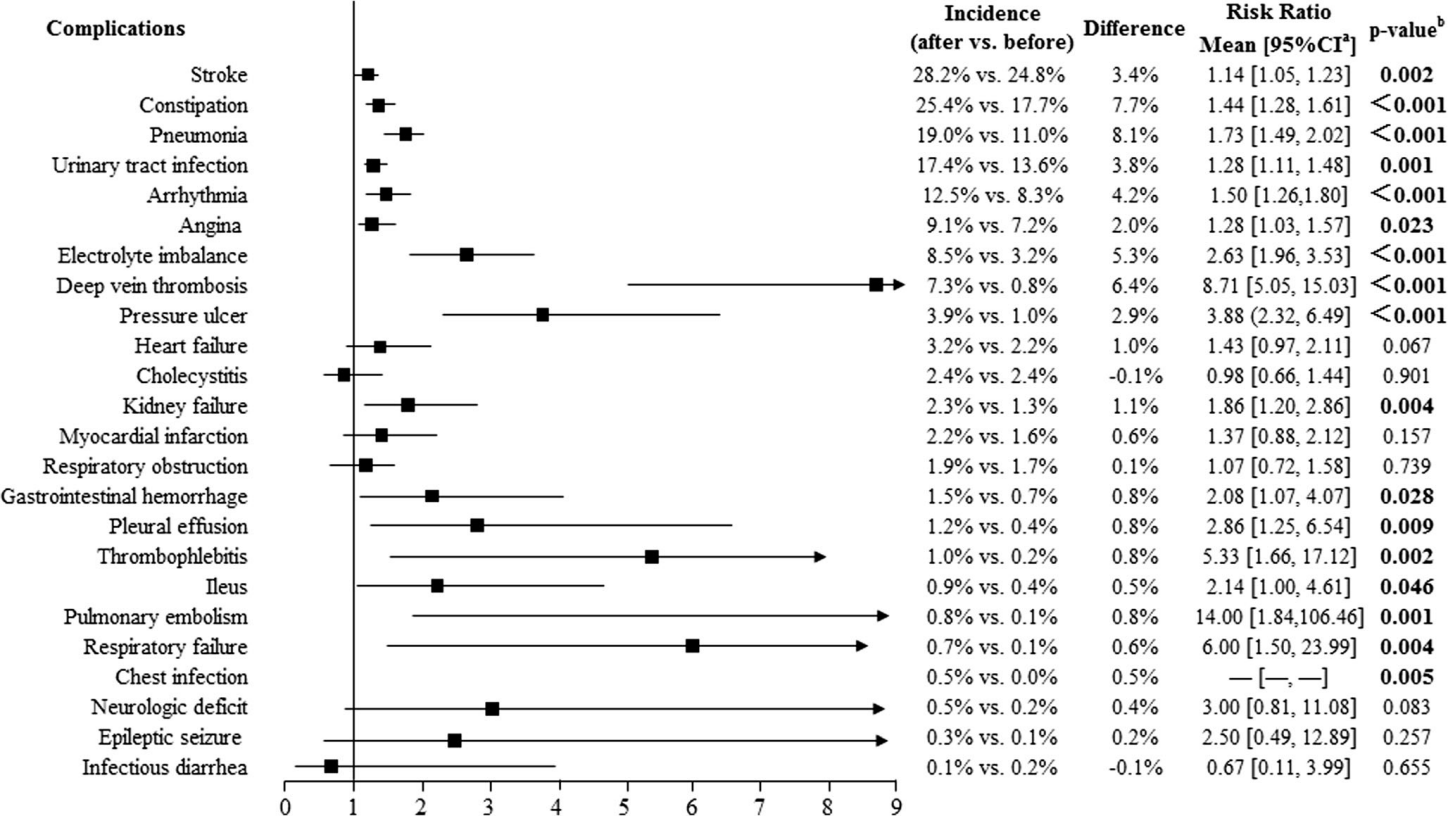
Accumulative incidence of medical complications within 12 months before and after fracture among hip fracture patients (*n* = 1675). The accumulative incidence of temporary respiratory insufficiency, diverticulitis, pulmonary edema, and infection of the central venous catheter was equal to zero, both in the 12 months before and after the index date. Difference = the incidence of complications within 12 months after fracture − the incidence of complications with 12 months before fracture. ^a^95% confidence interval (CI) of the risk ratio. ^b^Calculated using McNemar’s chi-squared test

**Fig. 3:**
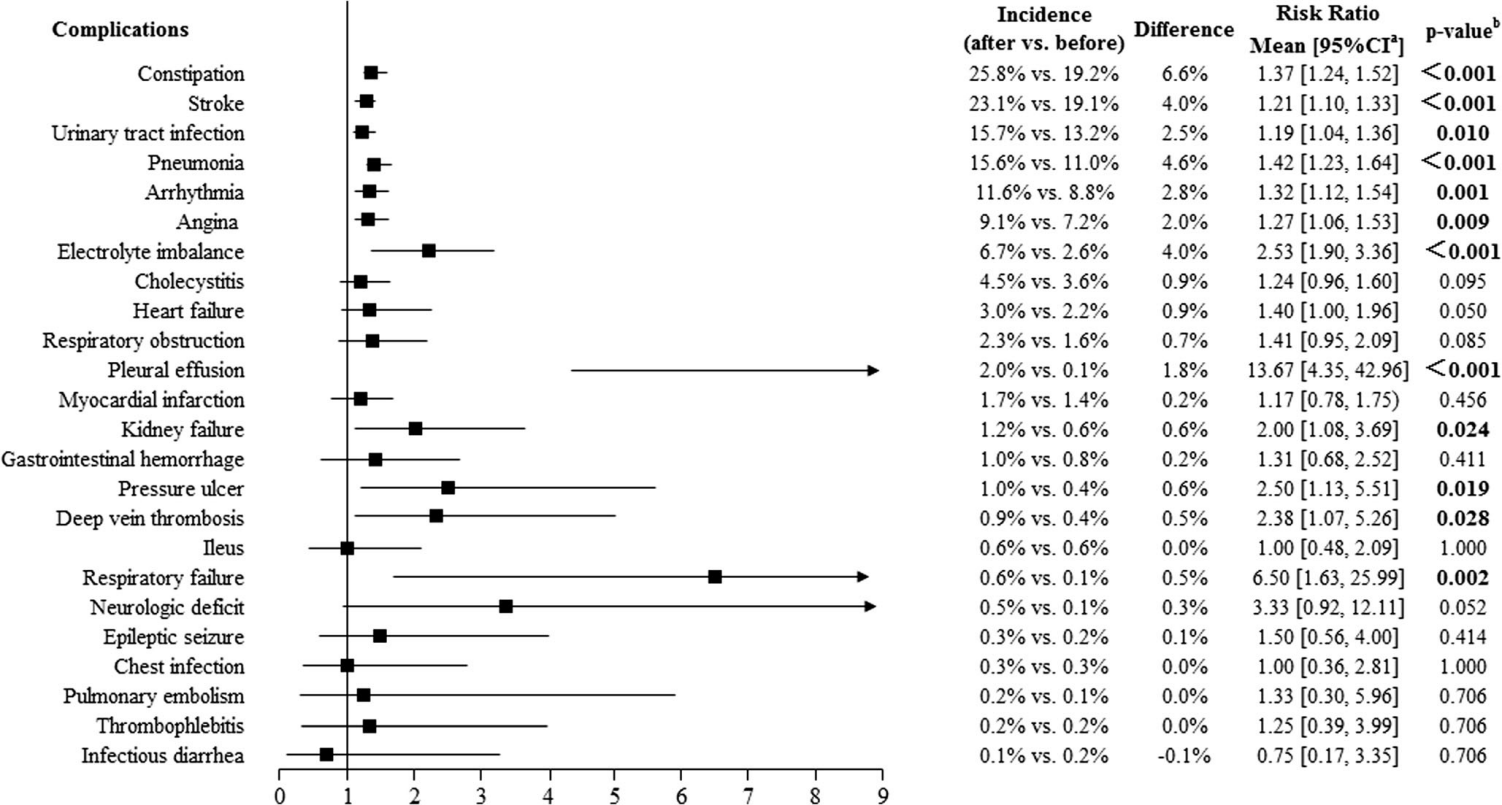
Accumulative incidence of medical complications within 12 months before and after fracture among vertebral fracture patients (*n* = 2089). The accumulative incidence of temporary respiratory insufficiency, diverticulitis, pulmonary edema, and infection of the central venous catheter was equal to zero, both in the 12 months before and after the index date. Difference = the incidence of complications within 12 months after fracture − the incidence of complications with 12 months before fracture. ^a^95% confidence interval (CI) of the risk ratio. ^b^Calculated using McNemar’s chi-squared test

For a comprehensive analysis of medical complications following osteoporotic fracture, the study identified medical complications by summarizing published prospective or review studies [13, 19–23], and a total of 28 complications (as showed in Figs. 1, 2, and 3) were included. The incidences of each medical complication were calculated for the 12 months before and the 12 months after the index osteoporotic fracture, respectively. The incidences within these two periods were compared. The incidences of medical complications were analyzed among total osteoporotic fracture samples as well as for the hip fracture group and vertebral fracture group separately.

The factors that were tested for association with the occurrence of medical complications included demographic characteristics (age, sex, and work status), fracture site (hip or vertebral), comorbidities, osteoporosis-related drug use history, and baseline direct medical cost. Comorbidities included hypertension, hyperlipoidemia, chronic heart disease (including arrhythmia, coronary heart disease, angina, myocardial infarction, and congestive heart failure), cerebrovascular disease, diabetes, chronic pulmonary disease, peripheral vascular disease, hemiplegia, kidney disease, peptic ulcer, rheumatic diseases, liver disease, dementia, and Parkinson’s disease. The Charlson Comorbidity Index (CCI) was calculated to evaluate patients’ disease history at baseline and to describe the number and percentage of patients with comorbidities. Osteoporosis-related drug use history, defined as any prescription of antiosteoporosis drugs at baseline, including calcium, vitamin D, active vitamin D and its analogs, calcitonin, bisphosphonates, estrogen, and selective estrogen receptor modulators, was also included [24].

Direct medical costs were calculated and estimated within the 12- and 24-month follow-up periods and represented the sum of direct medical costs related to all the healthcare services occurring during the follow-up periods. Direct medical costs were classified as osteoporosis-related direct medical costs and all-cause direct medical costs. Osteoporosis-related direct medical costs were identified and calculated when the primary or secondary diagnosis was osteoporosis and related fractures, whereas the all-cause direct medical costs referred to all expenses incurred during the follow-up periods. Subgroup analyses were conducted for osteoporosis-related and all-cause direct medical costs among patients with at least one medical complication and without any medical complications.

### Statistical analysis

Descriptive statistical methods were used to produce a profile of patients’ baseline characteristics, accumulative incidence of medical complications, and direct medical costs, with continuous variables reported using means and standard deviations (SDs) and categorical variables reported using absolute frequencies and percentages. The comparison of medical complication incidences within 12 months before and after the index fracture was conducted using McNemar’s chi-squared test. For the subgroup analysis of direct medical costs, Student’s *t* test was used for continuous variables, whereas chi-squared test was used for category variables. Finally, a logistic regression model was used to explore the risk factors of medical complications.

All statistical analyses were performed using STATA software (version 12.0; StataCorp LLC, College Station, TX, USA). The statistical significance level was defined as a two-sided alpha of 0.05.

## Results

### Patients’ baseline characteristics

As shown in selection flow chart (Supplementary Fig. 1), there are 7489 patients who were diagnosed with hip or vertebral fracture during January 1, 2010 to December 31, 2012. After ruling out those patients aged < 50 years old, with cancer, malignancy, multiple myeloma, or Paget’s disease of bone during the whole study period or without continuous enrollment and complete cost information, a total of 3719 patients with hip or vertebral fracture were included in this analysis. Patients’ baseline characteristics are shown in Table 1. Mean patient age was 68.4 ± 10.5 years, with most patients aged between 50 and 79 years (50–59 years, 25.6%; 60–69 years, 26.0%; and 70–79 years, 32.1%). Of the study patients, 62.4% were female and 90.4% were retired. Approximately 45% of the index fractures were hip fractures and 56.2% were vertebral fractures. Mean CCI was 1.34 ± 1.58, and chronic heart disease (56.5%), hypertension (55.8%), diabetes (27.8%), cerebrovascular disease (27.0%), hyperlipoidemia (26.5%), and chronic pulmonary disease (22.9%) were common conditions among these patients.

**Table 1:** Baseline demographic characteristics, healthcare resource utilization, and direct medical costs of osteoporotic fracture patients

	Total samples (*N* = 3719)	With complications (*n* = 2384)	Without complications (*n* = 1335)	*p* value^a^
Age, mean ± SD (years)	68.4 ± 10.5	69.7 ± 10.4	66.3 ± 10.2	< 0.001
Age, no. (%) (years)				< 0.001
50–59	951 (25.6)	515 (21.6)	436 (32.7)	
60–69	965 (26.0)	596 (25.0)	369 (27.6)	
70–79	1194 (32.1)	813 (34.1)	381 (28.5)	
≥ 80	609 (16.4)	460 (19.3)	149 (11.2)	
Female, no. (%)	2319 (62.4)	1462 (61.3)	857 (64.2)	0.083
Retired, no. (%)	3363 (90.4)	2203 (92.4)	1160 (86.9)	< 0.001
Fracture site, no. (%)				0.215
Hip	1675 (45.0)	1097 (46.0)	578 (43.3)	
Vertebral	2089 (56.2)	1324 (55.5)	765 (57.3)	
Charlson Comorbidity Index, mean ± SD	1.34 ± 1.58	1.61 ± 1.68	0.84 ± 1.22	< 0.001
Comorbidities, no. (%)				
Chronic heart disease^b^	2102 (56.5)	1555 (65.2)	547 (41.0)	< 0.001
Hypertension	2074 (55.8)	1539 (64.6)	535 (40.1)	< 0.001
Diabetes	1033 (27.8)	756 (31.7)	277 (20.7)	< 0.001
Cerebrovascular disease	1005 (27.0)	808 (33.9)	197 (14.8)	< 0.001
Hyperlipoidemia	984 (26.5)	747 (31.3)	237 (17.8)	< 0.001
Chronic pulmonary disease	852 (22.9)	629 (26.4)	223 (16.7)	< 0.001
Peripheral vascular disease	535 (14.4)	414 (17.4)	121 (9.1)	< 0.001
Hemiplegia	223 (6.0)	208 (8.7)	15 (1.1)	< 0.001
Kidney disease	229 (6.2)	171 (7.2)	58 (4.3)	0.001
Peptic ulcer	188 (5.1)	137 (5.7)	51 (3.8)	0.010
Rheumatic diseases	146 (3.9)	105 (4.4)	41 (3.1)	0.045
Liver disease	103 (2.8)	75 (3.1)	28 (2.1)	0.062
Dementia	42 (1.1)	36 (1.5)	6 (0.4)	0.003
Parkinson’s disease	32 (0.9)	29 (1.2)	3 (0.2)	0.002
Osteoporosis-related drug use^c^, no. (%)	644 (17.3)	459 (19.3)	185 (13.9)	< 0.001
All-cause hospitalizations, no. (%)	631 (17.0)	509 (21.4)	158 (11.8)	< 0.001
All-cause outpatient visits, no. (%)	3112 (83.7)	2109 (88.5)	1003 (75.1)	< 0.001
All-cause direct medical costs, mean ± SD ($)	1251 ± 2220	1513 ± 2456	784 ± 1621	< 0.001

^a^*p* value calculated using Student’s *t* test for continuous variables and chi-squared test for categorical variables

^b^Chronic heart disease includes arrhythmia, coronary heart disease, angina, myocardial infarction, and congestive heart failure

^c^Osteoporosis-related drugs include calcium, active vitamin D and its analogs, calcitonin, bisphosphonates, estrogen, vitamin D, and selective estrogen receptor modulators

A total of 2384 patients experienced medical complications during the 12 months following the index date. Compared with patients without complications, those with complications were older (69.7 ± 10.4 vs. 66.3 ± 10.2 years; *p* < 0.001), had a higher mean CCI (1.61 ± 1.68 vs. 0.84 ± 1.22 years; *p* < 0.001), more healthcare resource utilization, and higher direct medical costs at baseline (all *p* < 0.001).

### Accumulative incidence of medical complications

Figure 1 provides detailed information on the accumulative incidence of medical complications among the overall samples. A proportion of 64.1% of patients experienced at least one medical complication within 12 months following an osteoporotic fracture. The most common medical complications after an osteoporotic fracture were constipation (25.6%), stroke (25.2%), pneumonia (17.0%), urinary tract infection (16.3%), and arrhythmia (11.8%). The incidence of most medical complications, such as constipation (25.6% after vs. 18.6% before; risk ratio [95% confidence interval (CI)] 1.38 [1.28, 1.48]; *p* < 0.001), stroke (25.2 vs. 21.7%; 1.16 [1.09, 1.24]; *p* < 0.001), pneumonia (17.0 vs. 10.9%; 1.55 [1.40, 1.73]; *p* < 0.001), urinary tract infection (16.3 vs. 13.3%; 1.23 [1.12, 1.36]; *p* < 0.001), arrhythmia (11.8 vs. 8.5%; 1.39 [1.23, 1.56]; *p* < 0.001), angina (9.1 vs. 7.1%; 1.28 [1.11, 1.47]; *p* = 0.001), electrolyte imbalance (7.4 vs. 2.9%; 2.55 [2.07, 3.13]; *p* < 0.001), deep venous thrombosis (DVT; 3.7 vs. 0.6%; 6.27 [4.03, 9.71]; *p* < 0.001), heart failure (3.0 vs. 2.2%; 1.41 [1.09, 1.83]; *p* = 0.008), was significantly higher within 12 months after fracture than within 12 months before the fracture (all *p* < 0.05). The accumulative incidence of diverticulitis, temporary respiratory insufficiency, pulmonary edema, and infection of the central venous catheter was equal to zero within 12 months before and after fracture.

The accumulative incidences of medical complications for the hip fracture and vertebral fracture cohorts are shown in Figs. 2 and 3, respectively. The most common medical complications within 12 months after a hip or vertebral fracture were the same as those in the total osteoporotic fracture samples. Additionally, the incidences of gastrointestinal hemorrhage (1.5 vs. 0.7%; 2.08 [1.07, 4.07]; *p* = 0.028), thrombophlebitis (1.0 vs. 0.2%; 5.33 [1.66, 17.12]; *p* = 0.002), ileus (0.9 vs. 0.4%; 2.14 [1.00, 4.61]; *p* = 0.046), pulmonary embolism (0.8 vs. 0.1%; 14.00 [1.84, 106.46]; *p* = 0.001), and chest infection (0.5 vs. 0.0%; *p* = 0.005) were significantly higher after hip fracture than before. There was no significant difference in the incidence of these medical complications before and after a vertebral fracture. In addition, the incidence of DVT after fracture was 7.3% among hip fracture patients and 0.9% among vertebral fracture patients.

### Risk factors of medical complications

The results of multivariate logistic regression analysis are shown in Table 2. Compared with the 50–59-year-old cohort, 70–79-year-old patients (1.07 [1.00, 1.15]; *p* = 0.047) and > 80-year-old patients (1.17 [1.10, 1.25]; *p* < 0.001) showed a significantly higher risk of complications, whereas 60–69-year-old patients did not present a significantly higher risk. Interestingly, female patients (0.81 [0.69, 0.95]; *p* = 0.011) had a lower risk of complications compared with male patients. Multivariate analysis also revealed that retirement (1.38 [1.04, 1.84]; *p* = 0.026), history of hypertension (1.40 [1.15, 1.70]; *p* = 0.001), chronic heart disease (1.34 [1.10, 1.64]; *p* = 0.004), cerebrovascular disease (1.46 [1.19, 1.79]; *p* < 0.001), hemiplegia (3.74 [2.15, 6.52]; *p* < 0.001), Parkinson’s disease (3.65 [1.08, 12.28]; *p* = 0.037), and higher direct medical costs at baseline (1.07 [1.00, 1.14]; *p* = 0.037) were independent risk factors of complications.

**Table 2:** Multivariate logistic regression analysis of effect of baseline variables on incidence of medical complications after osteoporotic fracture (*N* = 3719)

	Odds ratio	95% CI^a^	*p* value
Age (vs. 50–59 years)			
60–69 years	1.04	0.94, 1.16	0.441
70–79 years	1.07	1.00, 1.15	0.047
≥ 80 years	1.17	1.10, 1.25	< 0.001
Female (vs. male)	0.81	0.69, 0.95	0.011
Retired (vs. working)	1.38	1.04, 1.84	0.026
Fracture site (hip vs. vertebral fracture)	0.97	0.83, 1.13	0.692
Comorbidities (yes vs. no)			
Hypertension	1.40	1.15, 1.70	0.001
Hyperlipoidemia	1.06	0.87, 1.29	0.559
Chronic heart disease	1.34	1.10, 1.64	0.004
Cerebrovascular disease	1.46	1.19, 1.79	< 0.001
Chronic pulmonary disease	1.13	0.93, 1.36	0.218
Peripheral vascular disease	1.17	0.92, 1.49	0.203
Diabetes	1.00	0.83, 1.20	0.977
Hemiplegia	3.74	2.15, 6.52	< 0.001
Kidney disease	1.10	0.79, 1.52	0.569
Peptic ulcer	1.02	0.72, 1.44	0.927
Rheumatic diseases	1.05	0.71, 1.55	0.799
Liver disease	1.14	0.72, 1.82	0.568
Dementia	1.47	0.58, 3.71	0.413
Parkinson’s disease	3.65	1.08, 12.28	0.037
Osteoporosis-related drug use history (yes vs. no)	1.06	0.86, 1.30	0.590
Any baseline all-cause outpatient visits (yes vs. no)	0.81	0.52, 1.27	0.366
Any baseline all-cause inpatient visits (yes vs. no)	0.85	0.66, 1.09	0.199
Total baseline all-cause direct medical costs, mean ± SD	1.07	1.00, 1.14	0.037

^a^95% confidence interval (CI) of the odds ratio

### Direct medical costs

For total osteoporotic fracture samples, the mean ± SD total osteoporosis-related direct medical costs during the 12- and 24-month follow-up periods were $2515 ± $3772 and $2699 ± $3972, respectively, and total mean ± SD all-cause direct medical costs were $3913 ± $4812 and $5665 ± $6818, respectively (Supplementary Fig. 2).

The results of subgroup analyses for direct medical costs by cohort are shown in Table 3. Compared with patients without complications, those with complications had higher osteoporosis-related mean ± SD total costs (12 months, $2708 ± $4031 vs. $2170 ± $3233; *p* < 0.001; 24 months, $2909 ± $4231 vs. $2325 ± $3432; *p* < 0.001), and the difference was reflected in inpatient costs (12 months, $2607 ± $4054 vs. $2069 ± $3260; *p* < 0.001; 24 months, $2777 ± $4236 vs. $2196 ± $3449; *p* < 0.001). The mean ± SD all-cause total costs for patients with complications were significantly higher than those for patients without complications (12 months, $4570 ± $5360 vs. $2738 ± $3326; *p* < 0.001; 24 months, $6700 ± $7721 vs. $3815 ± $4208; *p* < 0.001).

**Table 3:** Average direct medical cost per osteoporotic fracture patient^a^

	With complications (*n* = 2384)	Without complications (*n* = 1335)	*p* value^b^
Osteoporosis-related direct medical cost, mean ± SD ($)			
Accumulative total cost			
12 months	2708 ± 4031	2170 ± 3233	< 0.001
24 months	2909 ± 4231	2325 ± 3432	< 0.001
Accumulative inpatient cost			
12 months	2607 ± 4054	2069 ± 3260	< 0.001
24 months	2777 ± 4236	2196 ± 3449	< 0.001
Accumulative outpatient cost			
12 months	101 ± 151	101 ± 168	0.980
24 months	131 ± 243	128 ± 233	0.701
All-cause direct medical cost, mean ± SD ($)			
Accumulative total cost			
12 months	4570 ± 5360	2738 ± 3326	< 0.001
24 months	6700 ± 7721	3815 ± 4208	< 0.001
Accumulative inpatient cost			
12 months	3386 ± 5142	2145 ± 3309	< 0.001
24 months	4323 ± 6896	2562 ± 3790	< 0.001
Accumulative outpatient cost			
12 months	1184 ± 1524	593 ± 882	< 0.001
24 months	2377 ± 3070	1253 ± 1829	< 0.001

^a^Direct medical costs are the sum of the costs associated with all services, including drugs, medical consumables, laboratory and diagnostic tests, non-medication treatments, surgeries, daily room fees, and blood transfusions

^b^*p* value calculated using Student’s *t* test

## Discussion

To our knowledge, our study is the first retrospective database cohort study to use a large sample to analyze medical complications not necessarily directly related to a surgery and the first to identify risk factors and quantify direct medical costs following osteoporotic fracture in China. Among published studies, the categories of medical complications vary significantly because the definitions differ [10, 20]. To better evaluate the presence and types of medical complications after osteoporotic fracture and to gain a comprehensive result, we defined medical complications by summarizing published literature [13, 19–23], including four prospective studies [13, 19, 21, 22], one meta-analysis study [20], and one monograph [23].

After reviewing the existing literature, we found that a large majority of published research focused on the incidence of complications within a relatively short term after a fracture [9, 26, 27]. Especially, many studies on Chinese patients were restricted to complications within the perioperative period [17, 28–30]. Hence, given the current lack of data on the long-term complications rate after osteoporotic fracture, our study analyzed the incidence of medical complications within 1 year, which provided a broader picture for the prognoses of osteoporotic fracture patients over a longer period.

A difficulty in evaluating the risk of complications following a fracture using a claims database was to confirm whether a complication was related to osteoporotic fracture. We overcame this by analyzing medical complications within 12 months before and after an osteoporotic fracture and assessed the elevated risk by comparing the incidences between the two periods. This comparative method allowed us to use the prefracture period of a patient as a control for the postfracture period, thus accounting for any confounding factors that did not change within each patient. As far as we know, our study is the first to apply this comparison method. We also conducted a sensitivity analysis using a 4-month period instead of a 12-month period to ensure that the elevated complication risks were not mainly due to aging within 12 months. The sensitivity analysis showed similar trends (data not shown).

Our study showed that the most common medical complications within 12 months after fracture among osteoporotic hip or vertebral fracture patients were stroke, constipation, pneumonia, urinary tract infection, arrhythmia, angina, and electrolyte imbalance (Figs. 2 and 3). Lawrence et al. [11] reported that the most common inpatient complications were cardiac and pulmonary complications (8 and 4% of patients, respectively) among hip patients aged ≥ 60 years. Another study based on the clinical records of discharged hip fracture patients aged 70–94 years in China [15] suggested that the most common postoperative complications in 167 elderly patients with hip fracture were cardiac events (*n* = 37), DVT (*n* = 35), delirium (*n* = 25), pneumonia (*n* = 7), and stroke (*n* = 5). Yet another study reported that complications also correlate with the type and severity of patients’ comorbidities [14]. Compared with these studies, we had a longer follow-up period, which may show a higher rate for some complications due to the development of comorbidities.

A retrospective cross-sectional study by Sever et al. [25] indicated that the most common postoperative complications in patients with osteoporotic vertebral fracture were urinary tract infection (15.1%) and pressure ulcer (12.2%), and the incidence of pneumonia was only 4.4%. Another observational comparative study suggested that in-hospital complications following an osteoporotic vertebral compression fracture, including urinary tract infection (5%), pressure ulcer (3.75%), pneumonia (3.75%), and constipation (1.25%), were common in patients receiving conservative treatment, whereas no complications occurred in patients treated with vertebroplasty [26]. In addition to prefracture comorbidities and study duration, the differences in treatment selection and disease management also may result in the differences between our results and those in the published literature.

Previous literature suggests that DVT and pulmonary embolism are two of the most common and serious complications following fracture surgery, as well as the primary causes of death [15]. In our series, 7.3% of hip fracture patients experienced DVT, similar to the results of a prospective study by Soon et al. [10] that reported an 8.6% incidence of DVT following hip fracture surgery. The 12-month accumulative incidence of pulmonary embolism in our study was 0.8%. A consecutive annual cohort study of 664 patients by Hansson et al. [12] reported a 1 and 2% incidence of DVT and pulmonary embolism within 1 year after hip fracture, respectively. Our study showed that the accumulative incidence of DVT and pulmonary embolism among vertebral fracture patients was 0.9 and 0.2%, respectively. The lower incidence of DVT and pulmonary embolism among vertebral fracture patients has been demonstrated in previous studies [9, 25].

We identified age and diagnosis of hypertension, chronic heart disease, cerebrovascular disease, hemiplegia, or Parkinson’s disease at baseline as independent risk factors that predict complications after osteoporotic fracture. These results are supported by previous research [27–30]. Some previous studies suggested that diabetes contributed to the development of complications following hip fracture [28, 31], but it was not significant in our study. Moreover, our study showed that retired patients were at higher risk of complications, which can be interpreted as retired patients always being older. Furthermore, we also found that patients with higher baseline direct medical costs were more likely to develop complications after fracture. Currently, evidence of risk factors for complications in vertebral fracture is rare.

Finally, we assessed the accumulative direct medical costs of patients within 12 and 24 months after osteoporotic fracture. Total all-cause direct medical costs within 12 and 24 months were $3913 ± $4812 and $5665 ± $6818, respectively. A prospective observational data collection study published in 2014 suggested that the average direct medical care costs in western China were approximately RMB 17007 ($2699) per year per patient, which is lower than that in our study [6]. Our research showed that direct medical costs, especially osteoporosis related, within the first 12 months accounted for a vast majority. Subgroup analysis indicated that direct medical costs for patients with complications were significantly higher than those for patients without complications (all *p* < 0.001), which can be explained by the fact that complications can dramatically increase patients’ mean length of hospital stay [14].

Although the current study was carefully designed, the results must be interpreted within the context of the following limitations. First, we divided patients into subgroups depending on the presence or absence of complications and did not consider the severity of any complications, which may lead to bias in the results. Previous researchers differentiated complications using the Clavien-Dindo classification and found that most complications in geriatric hip fracture patients were grade II [21]. Second, we only identified the risk factors and evaluated the direct medical costs associated with any complication. Further studies are needed if burden and risk factor results related to a specific complication are desired. Third, the variables of height, weight, and smoking and drinking status, which may be related to the incidence of complications, cannot be identified in the UEBMI database. Studies have demonstrated that body mass index is associated with cardiac events and non-cardiac medical complications after hip fracture [30, 32]. Fourth, this study only included patients with continuous enrollment during 24 months after the index date and excluded those without continuous enrollment. Exact dates and reasons for insurance discontinuation, whether due to death, relocation, insurance cancelation, or any other reasons, were not available in the UEBMI database. By only including the insurance maintainers within 24 months post fracture, there may be underestimation in the incidence of medical complications and direct medical costs among patients with osteoporotic fractures.

This study was based on the UEBMI claims data in Tianjin, one of the four municipal cities and a typical tier-2 city of China. As the database mainly includes urban population, the generalizability of our findings to other, especially rural, areas of China may be limited. Among the research findings, direct medical costs may vary across developed and less-developed regions in China, but our findings on direct medical costs can reflect the economic burden of Chinese urban patients with osteoporotic fractures to some degree. For complication incidences, the variation across regions is considered to be relatively small, and therefore, the finding that incidence of medical complication increased after an osteoporotic fracture should be generalizable to other areas of China. Without national-level long-term data available in China, the findings of the present study could be considered as an important reference for the disease management among Chinese osteoporotic fracture patients.

## Conclusions

The incidences of a number of medical complications increased significantly among patients after osteoporotic fracture. Common medical complications following osteoporotic fracture include constipation, stroke, pneumonia, urinary tract infection, arrhythmia, angina, and electrolyte imbalance. Medical complications following osteoporotic fracture were associated with increased direct medical costs. Advanced age, male sex, retirement status, baseline comorbidities, and baseline direct medical costs were associated with the occurrence of medical complications following osteoporotic fracture. Effective clinical strategies should be used to prevent osteoporotic fractures and their complications, especially for those at high risk of complications.

## Electronic supplementary material

ESM 1 (DOCX 123 kb)
