# Supplementary material for: Incidence and risk factors of medical complications and direct medical costs after osteoporotic fracture among patients in China
Source: Arch Osteoporos. 2018 Feb 27;13(1):12. doi: 10.1007/s11657-018-0429-5 (PMC5829109; doi:10.1007/s11657-018-0429-5)
Supplement: Supplementary file 1 — (DOCX 123 kb) [file 11657_2018_429_MOESM1_ESM.docx]

**Supplementary material**

**Osteoporosis International**

**Incidence and Risk Factors of Medical Complications and Direct Medical Costs after Osteoporotic Fracture Among Patients in China**

Ruiqi Liu^1^, Aijun Chao^2^, Ke Wang^3^, Jing Wu^1^

^1^School of Pharmaceutical Science and Technology, Tianjin University, Tianjin 300072, China

^2^Tianjin Hospital, Tianjin 300072, China

^3^Lilly Suzhou Pharmaceutical Co. Ltd, Shanghai 200021, China

**Address correspondence to:** Jing Wu, School of Pharmaceutical Science and Technology, Tianjin University, No. 92 Weijin Rd., Nankai District, Tianjin, P.R. China, 300072; E-mail: [jingwu@tju.edu.cn](mailto:jingwu@tju.edu.cn)

**Conflict of Interest**

Ruiqi Liu, Aijun Chao, Ke Wang, and Jing Wu declare that they have no conflict of interest.

**Supplementary Figure 1:** Sample selection flow chart of osteoporosis-related fracture including hip and vertebral fracture

30% random sample of UEBMI enrollees in 2010-2012

N_1_=6,136,434×30%

Had at least one diagnosis of osteoporotic hip or vertebral fracture from Jan.1 2010 to Dec.31 2012

N_2_= 7,489

Patients aged ≥50 years old at index date N_3_= 6,371

Patients with continuous enrollment during the 12 months before and 24 months after index date

N_5_= 5,436

Patients without cancer/malignancy, multiple myeloma, and Paget’s disease of bone between 2009 and 2014

N_4_=4,881

Patients newly diagnosed with hip or vertebral fracture at index date

N=3,719

Excluded patients with history of fracture in 12 months before index date

As shown in this selection flow chart, there are 7,489 patients had the diagnoses of osteoporotic hip or vertebral fracture from January 1, 2010 to December 12, 2012, while 5,436 patients aged ≥50 years old and had continuous enrollment during the 12 months before and 24 months after index date. After excluding those patients developed cancer, malignancy, multiple myeloma, or Paget’s disease of bone during the whole study period, there are 4,881 remaining patients. Moreover, patients with history of fracture in 12 months before index date were excluded from this study. So 3,719 patients newly diagnosed with osteoporotic hip or vertebral fracture were included in this analysis finally.

**Supplementary Figure 2:** Average direct medical cost per patient of total osteoporotic fracture samples ($)

The figure shows the direct medical costs of total osteoporotic fracture samples including the osteoporosis-related and all cause direct medical costs during 12- and 24-month follow up period.
